# Contribution of the Ade Resistance-Nodulation-Cell Division-Type Efflux Pumps to Fitness and Pathogenesis of *Acinetobacter baumannii*

**DOI:** 10.1128/mBio.00697-16

**Published:** 2016-05-31

**Authors:** Eun-Jeong Yoon, Viviane Balloy, Laurence Fiette, Michel Chignard, Patrice Courvalin, Catherine Grillot-Courvalin

**Affiliations:** a Institut Pasteur, Unité des Agents Antibactériens, Paris, France; b INSERM, UMR S 938, CDR Saint-Antoine, Paris, France; c Sorbonne Université, UPMC, CDR Saint-Antoine, Paris, France; d Institut Pasteur Unité Histopathologie Humaine et Modèles Animaux, Paris, France

## Abstract

Overexpression of chromosomal resistance-nodulation-cell division (RND)-type efflux systems with broad substrate specificity contributes to multidrug resistance (MDR) in *Acinetobacter baumannii*. We have shown that modulation of expression of the structural genes for the efflux systems AdeABC and AdeIJK confers MDR and results in numerous alterations of membrane-associated cellular functions, in particular biofilm formation. However, the contribution of these RND pumps to cell fitness and virulence has not yet been studied. The biological cost of an antibiotic resistance mechanism is a key parameter in determining its stability and dissemination. From an entirely sequenced susceptible clinical isolate, we have generated a set of isogenic derivatives having single point mutations resulting in overexpression of each efflux system or with every pump deleted by allelic replacement. We found that overproduction of the pumps results in a significant decrease in fitness of the bacterial host when measured by competition experiments *in vitro*. Fitness and virulence were also evaluated *in vivo* both in systemic and pulmonary infection models in immunocompetent mice. A diminished competitiveness of the AdeABC-overexpressing mutant was observed only after intraperitoneal inoculation, but not after intranasal inoculation, the latter mimicking the most frequent type of human *A. baumannii* infection. However, in mice infected intranasally, this mutant was more virulent and stimulated an enhanced neutrophil activation in the lungs. Altogether, these data account for the observation that *adeABC* overexpression is common in MDR *A. baumannii* frequently found in ventilator-associated pneumonia.

## IMPORTANCE

Overproduction of the RND AdeABC efflux system is observed with a high incidence in multidrug-resistant *Acinetobacter baumannii* and results in increased resistance to several antibiotics of choice for the treatment of infections caused by this nosocomial pathogen. It was therefore important to study the biological cost of the overexpression of the *adeABC* structural operon which is normally tightly regulated. Fitness diminution of an overexpressing mutant detected *in vitro* and *in vivo* in a model that mimics sepsis was not observed in a pulmonary infection model in which the mutant was more virulent. This points out that increased virulence can occur independently from prolonged persistence in the infected organ and can account for the elevated incidence of this resistance mechanism in clinical isolates. The study also indicates that transposon libraries will identify only virulence genes that are expressed under physiological conditions but not those that are tightly regulated.

## INTRODUCTION

*Acinetobacter baumannii* is a major nosocomial pathogen that is clinically and epidemiologically successful at least in part due to its ability to persist in the hospital environment (1). Multidrug-resistant (MDR) strains have recently emerged resulting from the high capacity of *A. baumannii* to acquire genetic determinants and to the overproduction of resistance-nodulation-cell division (RND) efflux systems with broad substrate specificities (2). Three *Acinetobacter* drug efflux (Ade) RND systems, AdeABC (3), AdeFGH (4), and AdeIJK (5), have been reported to contribute to MDR. Expression of each pump is tightly regulated, AdeABC by the two-component regulatory system AdeRS (6), AdeFGH by the LysR-type transcriptional regulator AdeL (4), and AdeIJK by the TetR transcriptional regulator AdeN (7). Overexpression of the *adeABC* operon secondary to mutations in AdeRS plays a major role in MDR of clinical isolates (6, 8–10), whereas overexpression of *adeFGH* is rarely observed (8, 10); the constitutive expression of *adeIJK* is associated with intrinsic resistance (5, 9).

The fitness cost of antibiotic resistance mechanisms is a key parameter in determining the success of resistant bacteria (11). It is well established that efflux systems extrude not only antibiotics but also intracellular metabolites and that they play a role in the pathogenicity of Gram-negative bacteria (12). Inactivation of genes for efflux pumps impairs colonization or virulence as reported for *acrAB* in *Salmonella enterica* serovar Typhimurium (13), *mexAB-oprM* in *Pseudomonas aeruginosa* (14), *acrAB* in *Klebsiella pneumoniae* (15), and *acrAB-tolC* disruption in *Enterobacter cloacae* reduces both bacterial fitness and host virulence (16). Overexpression of efflux pumps has various and contrasting consequences on cell fitness and virulence: MtrCDE overproduction in *Neisseria gonorrhoeae* leads to increased fitness *in vitro* and higher virulence in the mouse vaginal infection model (17), whereas in *Stenotrophomonas maltophilia*, SmeDEF overproduction impairs fitness and decrease virulence (18); MexEF-OprN overproduction does not alter the fitness of *Pseudomonas aeruginosa* (19).

We have shown that modulation of expression of the structural genes for the efflux systems AdeABC and AdeIJK results in numerous alterations of membrane-associated cellular functions, in particular biofilm formation (9). However, the involvement of these RND systems in cell fitness and virulence has not yet been studied.

Several animal models are available to study virulence of clinical strains in terms of mortality, tissue bacterial load, histological score, and inflammatory cytokine levels (20). The most frequent *A. baumannii* nosocomial infections are ventilator-associated pneumonia, and a mouse model of pneumonia is obtained either through instillation of a bacterial suspension into the trachea (21) or intranasally (i.n.) (22, 23). The intraperitoneal (i.p.) route of infection is generally chosen to mimic systemic *A. baumannii* infections (24–26). While several investigations have been carried out using either of these experimental models, there are no reports of systematic comparison of both routes of infection with the same isolate.

In this study, the contribution of the three Ade RND systems on the fitness and virulence of *A. baumannii* was determined using an entirely sequenced susceptible clinical isolate and a set of isogenic derivatives having single point mutations resulting in overexpression of each of the efflux systems or with deletion of every pump by allelic replacement (9). We found that overexpression of the pumps results in a significant decrease in fitness of the bacterial host when measured by *in vitro* competition experiments. However, *in vivo* in systemic and pulmonary infection models in immunocompetent mice, there were different patterns of fitness and virulence. Altogether, these data account for the observation that MDR *A. baumannii* with *adeABC* overexpression are frequent in clinical settings.

## RESULTS

### Impact of overexpression or deletion of RND efflux systems on *in vitro* fitness of *A. baumannii*.

Growth rates were determined in monocultures at the beginning of the exponential phase in the absence of antibiotics, and the relative growth rates of the pump mutants are represented relative to that of the *A. baumannii* BM4587 parental strain (Table 1).

**TABLE 1:** Relative growth rates of *A. baumannii* BM4587 and derivatives overexpressing each pump or with each pump deleted

*A. baumannii* strain	Mutation^a^	Pump overexpression rate (fold)^a^^,^^b^	Relative growth rate (min^−1^)^c^	Reference
BM4587	Wild-type	1	1.00	33
BM4688	AdeS_R152K_	38 for *adeB*	0.96 ± 0.05	9
BM4689	AdeR_A91V_	222 for *adeB*	0.91* ± 0.04	9
BM4717	Δ*adeB*	NA	1.00 ± 0.03	9
BM4690	AdeL_N334H_	402 for *adeG*	0.82* ± 0.02	9
BM4691	AdeL_Q332stop_	636 for *adeG*	0.81* ± 0.03	9
BM4718	Δ*adeG*	NA	1.00 ± 0.03	9
BM4666	*adeN*_ΔC584_	6 for *adeJ*	0.94* ± 0.03	33
BM4719	Δ*adeJ*	NA	0.98 ± 0.01	9

a Data taken from reference 9.

b The pump overexpression rate values are shown as the fold or number of times the strain overexpressed the indicated gene. For instance, strain BM4688 overexpressed *adeB* 38-fold compared to the wild-type or parental BM4587 strain. NA, not applicable.

c The growth rates of derivatives overexpressing each RND efflux system or with each efflux system deleted were determined at the beginning of the exponential phase. The relative growth rate represents the ratio of growth of an isogenic derivative to that of the parental (wild-type) strain taken as 1.00. Every isolate was tested in duplicate in four to six independent experiments, and the results are presented as means ± SD. The mean values that are significantly different (*P* < 0.05 by the Student *t* test) from the value for the parental strain are indicated by an asterisk.

The two AdeABC-overproducing isogenic mutants, strains BM4688 and BM4689, presented a moderately reduced relative growth rate which paralleled the level of overproduction of the pump (0.96 for 38-fold and 0.91 for 222-fold increased *adeB* expression, respectively). No relative growth rate reduction was observed for deletion mutant derivative BM4717.

Two AdeFGH-overproducing mutants, BM4690 and BM4691, had the largest fitness disadvantage (0.82 ± 0.02 and 0.81 ± 0.03, respectively) associated with very high levels (402- and 636-fold) of *adeG* expression. Deletion of the *adeG* gene did not affect the host fitness.

The BM4666 mutant overexpressing AdeIJK displayed a significantly diminished relative growth rate, 0.94 ± 0.03, although *adeJ* was overexpressed only 6-fold. For the deletion mutant BM4719, a slight growth rate reduction was observed, 0.98 ± 0.01.

**FIG 1:**
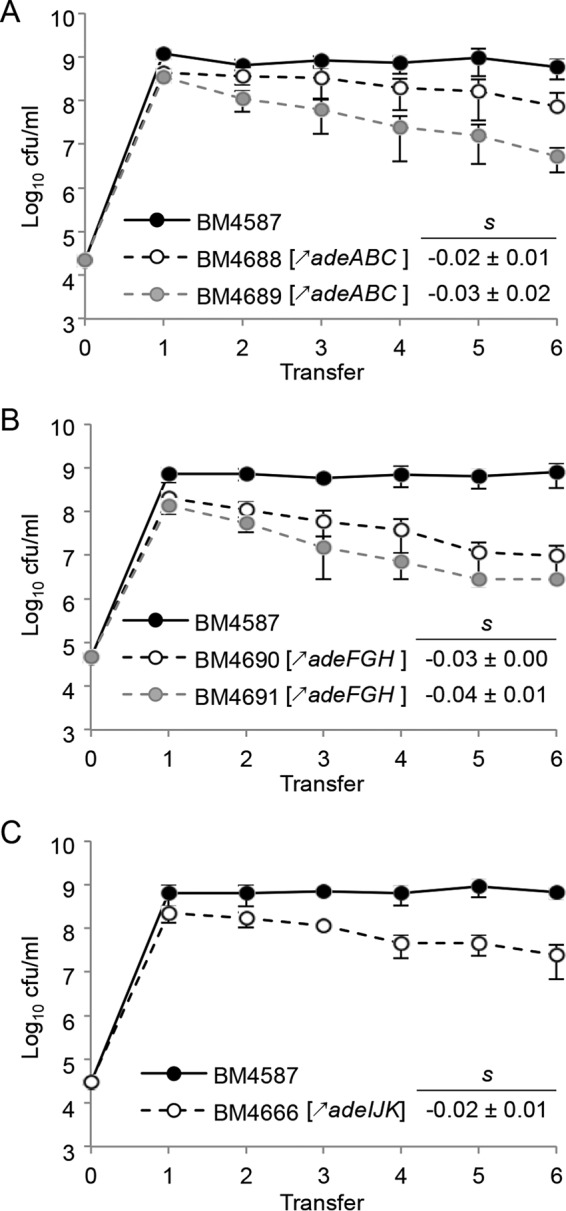
Growth competition between the parental strain and RND pump-overexpressing strains. (A) For *adeABC* overexpression, parental *A. baumannii* BM4587 strain and BM4688[↗*adeABC*] or BM4689[↗*adeABC*] were used. (B) For *adeFGH* overexpression, strains BM4587 and BM4690[↗*adeFGH*] or BM4691[↗*adeFGH*] were used. (C) For *adeIJK* overexpression, strains BM4587 and BM4666[↗*adeIJK*] were used. (A to C) For all experiments shown, the strains were mixed at a 1:1 ratio at an initial inoculum of 5 × 10^4^ CFU and transferred every 12 h (corresponding to approximately 20 generations) in fresh medium for up to six passages. The competition index (CI) was calculated as the CFU ratio of the resistant and susceptible strains (R/S) at time *t*_1_ divided by the same R/S at time *t*_0_, and the selection coefficient *s* was then calculated as the slope of the following linear regression model *s* = ln (CI)/[*t* × ln (2)], where *t* is the number of generations. Every experiment was carried out in du plicate in four independent experiments, and the results are presented as means ± SD (error bars).

For a more sensitive fitness evaluation, *in vitro* competition experiments were performed by mixing each derivative overexpressing a pump with the parent at an initial ratio of 1:1 for ca. 150 generations (Fig. 1). Strains BM4689[↗*adeABC*] (BM4689 overexpressing *adeABC*) and BM4688[↗*adeABC*] had competitive disadvantages of 3.1% and 2.2% per generation, respectively. Strains BM4691[↗*adeFGH*], BM4690[↗*adeFGH*], and BM4666[↗*adeIJK*] had competitive disadvantages of 4.3%, 2.7%, and 2.2% per generation, respectively.

### Comparison of *A. baumannii* virulence in mice after i.p. or i.n. inoculation.

The i.p. 50% lethal dose (LD_50_) of *A. baumannii* parental strain BM4587 was 4.9 × 10^5^ CFU/mouse, which indicates moderate virulence compared to other strains of *A. baumannii* with i.p. LD_50_ values in immunocompetent C57BL/6 mice ranging from 1.9 × 10^3^ to 7.8 × 10^6^ CFU (24, 25).

**FIG 2:**
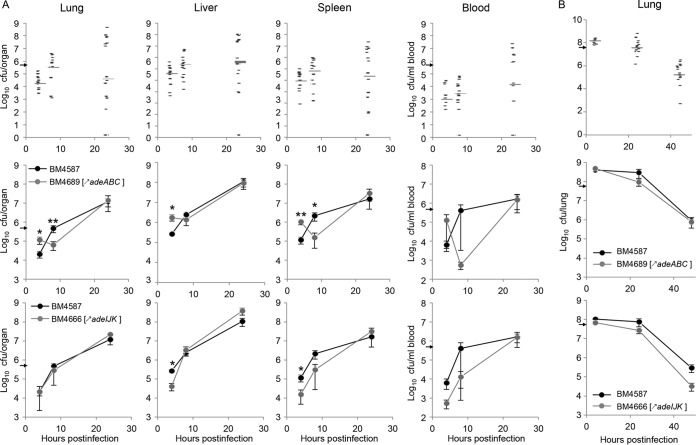
Bacterial loads in organs of mice infected with *A. baumannii* BM4587 and its derivatives. (A and B) C57BL/6 female mice were infected with *A. baumannii* strains given i.p. (A) or i.n. (B). (A) Mice infected i.p. were given 5 × 10^5^ CFU/mouse. Mice infected i.n. were given 5 × 10^7^ CFU/mouse. In the graphs in the top row in panels A and B, each symbol represents the value for an individual animal infected with strain BM4587 (17 mice infected i.p. and 16 mice infected i.n.), and the gray bar represents the median value of the group of mice. In the graphs in the middle and bottom rows in panels A and B, the means ± SE (error bars) of bacterial loads in organs of mice infected i.p. with either strain BM4689[↗*adeABC*] (13 mice infected i.p. and 6 mice infected i.n. [middle row]) or strain BM4666[↗*adeIJK*] (5 mice infected i.p. and 8 mice infected i.n. [bottom row]) and with parental strain BM4587 are indicated. The inoculating doses are indicated by small black arrows on the *y* axis. Values that are significantly different by two-tailed Mann-Whitney U test are indicated as follows: *, *P* < 0.05; **, *P* < 0.01.

Groups of mice were sacrificed at various time points after i.p. inoculation with ca. 5 × 10^5^ CFU/mouse of the parental strain and mutant strains, and bacterial loads in blood, lungs, liver, and spleen were quantified (Fig. 2A, top panels). In mice inoculated with parental strain BM4587, bacteria disseminated by 4 h from the peritoneum to blood (8.1 × 10^2^ CFU/ml [median values shown in parentheses]), lung (4 × 10^4^ CFU/lung), liver (3.2 × 10^5^ CFU/liver), and spleen (6.5 × 10^4^ CFU/spleen). Bacterial proliferation, of approximately 1 log_10_ unit, continued by 8 h with median values of CFU/organ of 2.5 × 10^3^ per ml of blood, 2.6 × 10^5^ per lung, 2.0 × 10^6^ per liver, and 5.1 × 10^5^ per spleen. Finally, by 24 h, mice either succumbed (less than 10% per experiment) or survived, and organ bacterial counts were very scattered, with median values of CFU/organ of 1.2 × 10^4^ per ml of blood, 3.2 × 10^4^ in lung, 4.0 × 10^6^ in liver, and 1.9 × 10^5^ in spleen.

By 4 h after i.p. injection of strain BM4689[↗*adeABC*], the bacterial load per organ increased between 2.3- and 8.4-fold compared to the bacterial load in the parent (Fig. 2A, middle panels). Bacterial multiplication then lagged by 8 h, and counts were significantly lower than those of strain BM4587. At 24 h postinoculation (p.i.), mean values were similar to those of the group infected with BM4587, as were the numbers of dead mice by 24 h. To further assess the early proliferative attenuation of BM4689[↗*adeABC*], randomly selected colonies obtained *ex vivo* at each time point were studied, but only nonsignificant differences in *in vitro* susceptibility tests and in growth rates were observed.

In the mice inoculated with strain BM4666[↗*adeIJK*], initial bacterial dissemination by 4 h to the liver and spleen was not as efficient as that of parental BM4587 with smaller bacterial burden in the spleen and liver. However, bacterial counts increased rapidly to reach similar levels by 24 h (Fig. 2A, bottom panels).

After i.n. inoculation of ca. 5 × 10^7^ CFU of strain BM4587, 1.2 × 10^8^ CFU/lung was recovered by 4 h and the CFU/lung gradually dropped with median values of 3.0 × 10^7^ by 24 h and 1.3 × 10^5^ by 48 h (Fig. 2B, top panel). The mice infected with BM4689[↗*adeABC*] or BM4666[↗*adeIJK*] displayed similar bacterial loads in the lungs, and the bacterial loads decreased at the same pace as in mice infected with parental BM4587 (Fig. 2B, middle and bottom panels). Extrapulmonary dissemination, except for occasional recovery in blood, was not observed at any time point.

### AdeABC overproduction induces a moderate competitive disadvantage in a systemic infection model.

**FIG 3:**
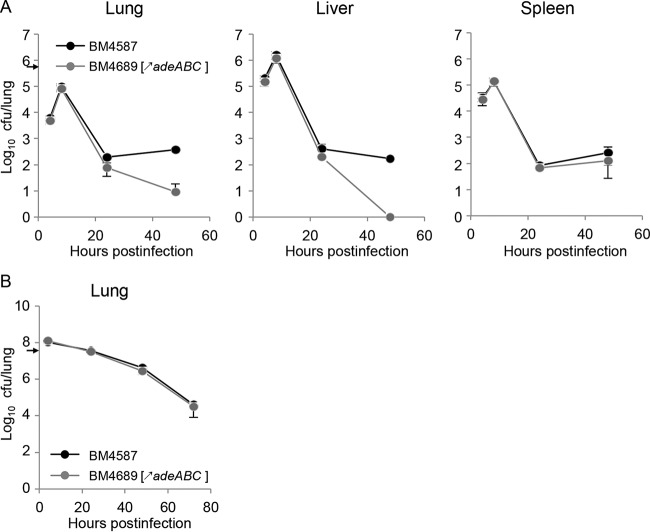
*In vivo* competition between strains BM4587 and BM4689[↗*adeABC*]. (A and B) *In vivo* competition was carried out in groups of six C57BL/6 female mice inoculated i.p. (A) or i.n. (B). Freshly cultured parent strain BM4587 and mutant strain BM4689[↗*adeABC*] were mixed at a 1:1 ratio to inoculate 5 × 10^5^ CFU/mouse for the i.p. route or 5 × 10^7^ CFU/mouse for the i.n. route. Values are means ± SD (error bars). The inoculating doses are indicated by small black arrows on the *y* axis.

Groups of six mice were inoculated with a 1:1 mixture of strains BM4587 and BM4689[↗*adeABC*] with ca. 5 × 10^5^ CFU/mouse for i.p. injection (Fig. 3A) and ca. 5 × 10^7^ CFU/mouse for i.n. inoculation (Fig. 3B), and bacterial loads in organs were monitored for 48 h and 72 h, respectively. After i.p. injection, the parental and mutant mixture kept the initial ratio by 8 h p.i. (Fig. 3A). By 24 h, slightly lower loads of BM4689[↗*adeABC*] were observed in the lungs and spleen (5.1 × 10^1^ CFU/lung and 6.7 × 10^1^ CFU/spleen, respectively) compared to BM4587 (2.1 × 10^2^ CFU/lung and 8.3 × 10^1^ CFU/spleen, respectively), and a continuous decrease in the relative loads was observed by 48 h (<10 CFU/lung, <10 CFU/liver, and 5.3 × 10^0^ CFU/spleen) compared to BM4587 (3.2 × 10^2^ CFU/lung, 2.0 × 10^2^ CFU/liver, and 6.2 × 10^1^ CFU/spleen). Strain BM4689[↗*adeABC*] was moderately less competitive than the parent during the course of systemic infection in agreement with the *in vitro* reduced growth rate and competitive disadvantage. In the lungs of mice infected by i.n. inoculation with a 1:1 mixture of BM4587 and BM4689[↗*adeABC*], the initial ratio remained constant during the entire experiment, up to 72 h (Fig. 3B), indicating no competitive disadvantage in the pulmonary infection model.

### Overproduction of AdeABC resulted in enhanced neutrophil activation after i.n. infection.

**FIG 4:**
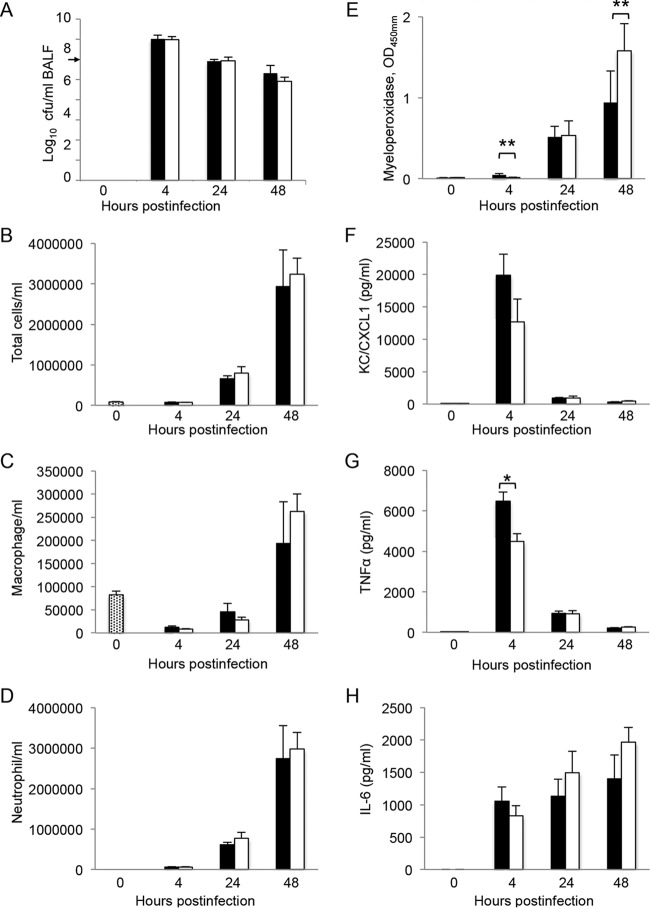
Bronchoalveolar lavage fluid analysis. BALF samples from groups of five C57BL/6 mice infected i.n. with 5 × 10^7^ CFU of strain BM4587 (black) or strain BM4689[↗*adeABC*] (white) were analyzed. (A) Bacteria in the BALF samples were quantified. (B to D) Total BALF cells/ml (B), macrophages (C), and neutrophils (D) were quantified by examining Hema 3-stained cytospin slides. (E to H) Myeloperoxidase was measured by enzyme immunometric assay (E), and proinflammatory cytokine/chemokine production was determined for KC/CXCL1 (F), TNF-α (G), and IL-6 (H) by using sandwich ELISA. Data are presented as means plus SD. Values that are significantly different by one-way ANOVA are indicated by bars and asterisks as follows: *, *P* < 0.05; **, *P* < 0.01.

To study the host immune response induced by pulmonary infections with strains BM4587 and BM4689[↗*adeABC*], bronchoalveolar lavage fluid (BALF) samples were collected from mice 4, 24, and 48 h after i.n. inoculation. Bacterial loads in BALF decreased steadily: from 1.2 × 10^7^ CFU/ml at 4 h to 9.1 × 10^5^ at 24 h, and 8.1 × 10^4^ at 48 h p.i. (Fig. 4A). Bacterial loads in BALF samples from mice infected with BM4689[↗*adeABC*] were indistinguishable from those of mice infected with BM4587.

Before infection, the number of total BALF cells was 8.2 × 10^4^ ± 1.8 × 10^4^, composed of only macrophages. After i.n. inoculation with strain BM4587, by 4 h the BALF cell number remained constant (7.4 × 10^4^ ± 2.3 × 10^4^ cells/ml) and was mainly composed of neutrophils (83% of total BALF cells). The number of cells increased from 6.6 × 10^5^ ± 1.5 × 10^5^ cells/ml by 24 h to 2.9 × 10^6^ ± 2.0 × 10^6^ by 48 h (more than 93% being neutrophils). Infection with BM4689[↗*adeABC*] induced similar changes in BALF composition. Despite the small difference in neutrophil populations between the groups infected with BM4587 or BM4689[↗*adeABC*], significant differences (*P* < 0.01) in the optical density at 450 nm (OD_450_) of myeloperoxidase (MPO), an enzyme with antimicrobial activity, between the groups were observed in particular at 48 h p.i., 0.9 ± 0.4 and 1.5 ± 0.3, respectively (Fig. 4E), indicating more-effective activation of neutrophils by BM4689[↗*adeABC*].

To further characterize the immune response induced by infection with the parental or mutant strains, we determined the levels of proinflammatory cytokines in the BALF samples (Fig. 4F to H). We observed substantial release of all cytokines by bacterial inoculation. In the BALF samples from mice infected with strain BM4587, the keratinocyte chemoattractant protein (KC)/chemokine (C-X-C motif) ligand 1 (CXCL1) peaked by 4 h (2.4 × 10^4^ ± 3.1 × 10^3^ pg/ml) and rapidly decreased by 24 h. The concentration of the released tumor necrosis factor alpha (TNF-α) also peaked at 4 h p.i. (6.4 × 10^3^ ± 3.6 × 10^2^ pg/ml) and decreased to 9.4 × 10^2^ ± 9.0 × 10^1^ pg/ml by 24 h. In the case of interleukin-6 (IL-6), the concentration increased moderately during infection. The kinetics of chemokine and cytokine production in BALF samples from mice infected with BM4689[↗*adeABC*] were similar, but the concentrations at 4 h p.i. of KC/CXCL1 and TNF-α were significantly lower than those elicited by BM4587 (Fig. 4F and G). The production of IL-6 was lower by 4 h but rapidly increased to concentrations higher than those obtained with the parent, although not reaching statistical significance (Fig. 4H).

After i.p. inoculation, BALF cells were composed of 99% pulmonary macrophages, and the levels of chemokines (the same panel of chemokines) were similar to what is found in noninfected animals. Bacterial loads in the BALF were under the detection threshold, whereas from 1.1 × 10^1^ to 2.0 × 10^3^ CFU/lung of bacteria was recovered. These results indicate that i.p. inoculation did not result in pulmonary infection and that the bacteria recovered in lungs after systemic infection were mostly from blood.

### Histopathological observations.

**FIG 5:**
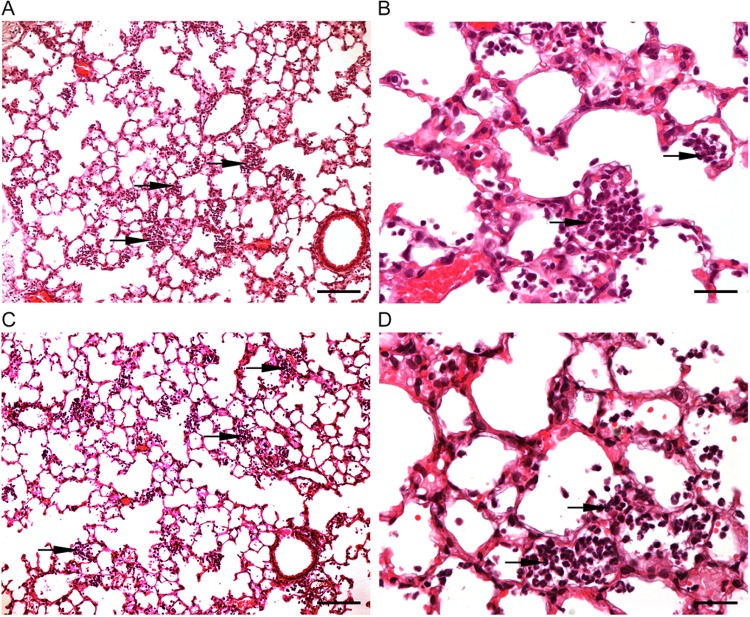
Histopathology of the lung in mice inoculated i.n. with *A. baumannii* BM4587 and BM468 9[↗*adeABC*]. (A to D) Paraffin-embedded lung sections stained with H&E from mice inoculated i.n. with 5 × 10^7^ CFU of *A. baumannii* BM4587 (A and C) or BM4689[↗*adeABC*] (B and D) at 24 h p.i. The black arrows point to foci of neutrophils. The original magnification was ×10. Bars, 100 µm (A and C) and 25 µm (B and D).

**FIG 6:**
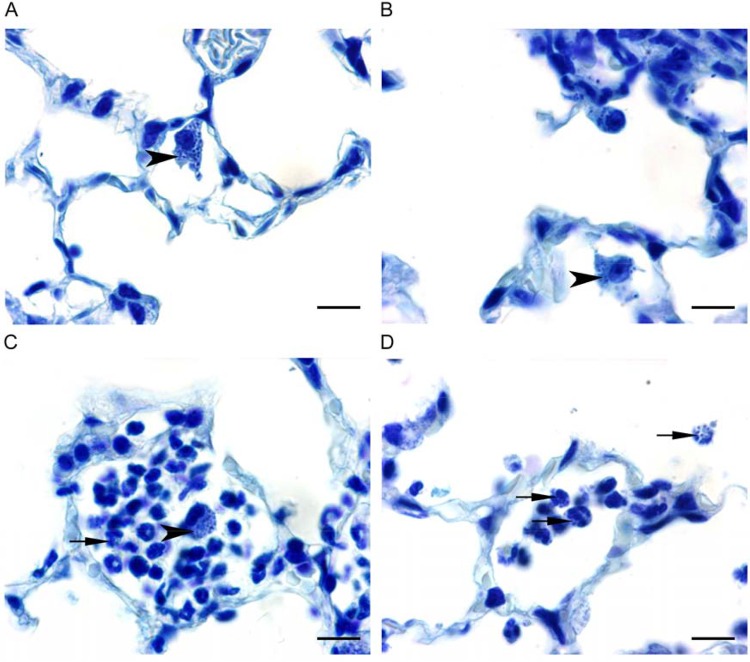
Toluidine blue-stained lung sections in mice inoculated i.n. with *A. baumannii* BM4587 and BM4689[↗*adeABC*]. (A to D) Paraffin-embedded sections of lungs stained with toluidine blue from mice inoculated i.n. with 5 × 10^7^ CFU of *A. baumannii* BM4587 (A and C) and BM4689[↗*adeABC*] (B and D) at 4 h p.i. (A and B) and 24 h p.i. (C and D). Bacteria appear as intracytoplasmic dark blue spots. The black arrows indicate infected neutrophils, and black arrowheads indicate infected macrophages.

In mice infected i.n. with strain BM4587, minimal neutrophil infiltration in alveolar lumens was observed by 4 h (data not shown). The severity of inflammatory lesions increased with time: multifocal mild neutrophil infiltrates were observed 24 h p.i. (Fig. 5), whereas moderate neutrophilic inflammation was noted after 48 h (data not shown). These microscopic changes were parallel and showed a similar time course in both groups inoculated either with strain BM4587 (Fig. 5A and B) or with strain BM4689[↗*adeABC*] (Fig. 5C and D). In addition, to assess the location of bacteria in the lesions, lung tissue sections were stained with toluidine blue. Very few bacteria were present in lungs 4 h p.i. with BM4689[↗*adeABC*] or BM4587. They were observed with both strains within alveolar macrophages and, very occasionally, in monocytes in blood vessel lumen or free in the alveolar lumen (Fig. 6A and B). Twenty-four hours p.i., bacteria were observed within macrophages and recruited neutrophils (Fig. 6C and D). More infected neutrophils were found in lungs of mice inoculated with BM4689[↗*adeABC*] compared to those inoculated with BM4587, corresponding to the elevated level of MPO in the BALF samples from mice infected with BM4689[↗*adeABC*]. After 48 h, bacteria were still present in macrophages and/or neutrophils in alveoli following inoculation with BM4587 or BM4689[↗*adeABC*]. No microscopic changes or bacteria were observed in the lung at any time point after i.p. inoculation of BM4689[↗*adeABC*] or BM4587. By inoculating by this route, we observed bacteria only in blood, and the bacteria were free or within vascular macrophages, in alveoli, or on the alveolar wall.

## DISCUSSION

Overexpression of chromosomal RND-type efflux systems with broad substrate specificity contributes to MDR in *A. baumannii* (9). Since such strains have emerged as a main concern in clinical settings, particularly in intensive care units, the role of RND pumps in antimicrobial resistance has been studied extensively but not their implications for virulence. We reported that overproduction of AdeABC is mainly responsible for MDR by efflux in clinical isolates (8). We have also generated, from an entirely sequenced susceptible *A. baumannii* clinical strain, a complete set of isogenic mutants overexpressing each of the three main RND systems or with each of the three main RND systems deleted and systematically evaluated the contribution of each Ade pump to antibiotic resistance and biofilm formation (9). We have shown that modulation of expression of the structural genes for these efflux systems led to several alterations in membrane-associated cellular functions, in particular overproduction of AdeABC and AdeIJK resulted in a decrease in biofilm formation (9). Therefore, a biological cost and an impact on virulence could be anticipated.

The fitness cost of a resistance determinant is best studied by comparison of isogenic strains differing only by this determinant (11). Of note, the levels of *adeB* expression in the mutants were in the same range as those in the 13 MDR clinical isolates (8). The biological cost observed for AdeABC overproduction was significant (2.2 to 3.1%; Fig. 1) but moderate compared to that associated with other resistance mechanisms, such as to colistin (8 to 20%**)** within the same species (27). The relatively low fitness burden associated with *adeABC* overexpression could be responsible, at least in part, for its high prevalence in MDR clinical isolates; in our study, 10 out of 13 MDR clinical isolates overexpressed *adeB* more than 20-fold (8). Among 444 clinical *A. baumannii* isolates, *adeB* overexpression was also the most common, with similar elevated levels, and was generally associated with an MDR phenotype (10). The *adeIJK*-overexpressing mutant had a relatively high fitness decrease (2.2%; Fig. 1). Such strains are found only occasionally: in a panel of 34 *A. baumannii* strains (28), a 3- to 4-fold increase in *adeJ* expression due to a truncated AdeN regulator was observed in three strains (unpublished data). In the study of 444 clinical *A. baumannii* isolates, the levels of *adeJ* expression in nine strains were ca. 10 times higher than the levels in the susceptible ATCC 17978 strain (10). Interestingly, strain BM4719[Δ*adeJ*] displayed a slightly diminished growth rate (0.98 ± 0.01), while the growth rates of the mutants with either *adeB* or *adeG* deleted were similar to that of the parent (Table 1), suggesting that constitutive expression of *adeIJK* is critical for *A. baumannii* physiology. The *adeFGH*-overexpressing mutant displayed the highest fitness cost (4.3%), but the expression rate of the system in this *in vitro* mutant was very high (Table 1), a level that has not been observed in clinical isolates (from 2- to 15-fold) (8, 10). Overall, there was good agreement between the burden imposed on the host by overexpression of an efflux system and its relative occurrence in clinical isolates. In the mutants, as well as in clinical strains, overexpression of the structural genes for the efflux pumps resulted from regulatory mutations. Thus, the biological cost could be due to excessive energy consumption by the pumps or too efficient export of molecules beneficial to the host. Alternatively, the regulatory genes may also control other genes that could be responsible for the fitness burden.

A recent approach to study fitness and virulence of pathogens is the use of saturated transposon (Tn) libraries to identify genes that contribute to optimal fitness and therefore virulence *in vivo*. As many as 157 *A. baumannii* genes required for persistence in a mouse pneumonia model were identified after i.n. injection of a transposon insertion library of strain ATCC 17978 (23). In the majority of cases, insertion led to a decrease in persistence. Insertion in two genes (A1S_1649 and A1S_1801) predicted to encode RND efflux pump determinants resulted in a decrease in the fitness of the mutants in mice (23). A similar result was obtained after i.p. injection of isogenic mutants of virulent strain AB5075 with Tn insertions in the same genes (26). However, none of these genes were related to the genes encoding the three components of the three efflux systems of our study. Growth analysis of a transposon library of AB5057 in the *Galleria mellonella* larva model detected 300 genes required for bacterial survival and identified the genes for AdeIJK (29). Of note, the *adeJ* deletion mutant was the only one with a slightly diminished growth rate, but unfortunately, it was not studied *in vivo*. Proteomic analysis of bacterial membranes indicated that, in susceptible strains, the AdeABC system is detected at very low levels, whereas all three proteins, AdeI, AdeJ, and AdeK, are easily detected in agreement with their basal constitutive synthesis (9). This observation points to the notion that the study of virulence using knockout transposons will identify only the genes that are expressed under the assay conditions. Genes with expression under tight regulation, such as *adeABC*, and the majority of the antibiotic resistance determinants will not be detected unless they are negatively regulated or when their induced expression results in a selective advantage under the experimental conditions used (30), which was not the case in this study.

The mouse pulmonary model is widely used to study *A. baumannii* virulence, although most isolates can induce only a self-limiting pneumonia with slow local bacterial multiplication. We have used both intraperitoneal and intranasal routes of infection with the same set of strains and observed a diminished competition of strain BM4689[↗*adeABC*] after i.p. inoculation, but not after i.n. inoculation (Fig. 3). However, the pneumonia model allows us to study the most frequent mode of infection in humans and to distinguish strains of various degrees of virulence and has revealed the important role of neutrophils, which are rapidly recruited for control of the respiratory infection (22). Only minor differences in bacterial growth kinetics were observed between parental and RND pump overexpression mutants (Fig. 2). However, BALF analysis and histopathological observations indicated an increased host defense response to BM4689[↗*adeABC*]. In BALF samples from mice infected with the mutant, a slight increase of neutrophils was observed 48 h p.i. (Fig. 4). TNF-α and KC/CLCX-1 contribute to neutrophil migration within the lung, while elevated IL-6 levels are a response to excessive inflammation (31). Increased MPO activity recovered in the BALF samples 48 h p.i. is the sign of neutrophil degranulation following activation (32). Histopathological observations showed that there were more infected neutrophils at 24 h after mutant infection (Fig. 5 and  6). Taken together, pulmonary infection by the *adeABC*-overexpressing mutant resulted in a small delay in the onset but in a more severe inflammation in the lung secondary to a higher activation of recruited neutrophils. These results indicate that persistence in the lung is dissociated from virulence, the latter being best appreciated by immunological responses of the host and histological analysis. This observation may explain at least in part, the success of *adeABC*-overexpressing MDR *A. baumannii* in ventilator-associated pneumonia.

## MATERIALS AND METHODS

### Bacterial strains and growth conditions.

Drug-susceptible parental *A. baumannii* BM4587 (33) and its mutant derivatives (9) are described in Table 1. Bacteria were grown in brain heart infusion (BHI), Luria-Bertani (LB), or Mueller-Hinton (MH) broth or agar at 37°C. Antibiotic susceptibility was assessed by disk diffusion on MH agar according to the CLSI guidelines (34).

### Determination of growth rates and growth competitions.

Growth rates were determined as described previously (35) in microplates coupled to a Multiscan spectrophotometer (Thermo Scientific). The strains were grown overnight, and the cultures were diluted to inoculate ~1 × 10^3^ bacteria into 200 µl of LB in a 96-well microplate which was incubated with shaking. Absorbance was measured at 595 nm every 3 min. Each culture was replicated three times in the same microplate. Growth rates, measured in five independent experiments, were determined at the beginning of the exponential phase, and relative growth rates were calculated as the ratio of the growth rate of the strains versus that of the parent. Competition experiments were performed in cocultures with 5 × 10^4^ of the susceptible strain and the resistant strain mixed in 10 ml of LB at an initial ratio of 1:1 and grown for 12 h (about 20 generations). The mixed culture was transferred to fresh LB by 10^6^-fold serial dilution every 12 h for up to six passages. The total number of viable cells was determined at the end of every transfer on nonselective plates, and the proportion of resistant strains was determined by replica plating 500 to 1,000 colonies on MH agar and MH agar supplemented with gentamicin (4 µg/ml and 12 µg/ml), clindamycin (200 µg/ml), or tetracycline (2 µg/ml), depending on the pair of strains studied. Each experiment was carried out in duplicate and performed four times independently.

### Mice.

Specific-pathogen-free female C57BL/6J mice were purchased from the Centre d’Elevage R. Janvier (Le Genest Saint-Isle, France) and were used at 6 to 8 weeks of age with a body weight of 17 to 19 g. The animals were housed under specific-pathogen-free conditions in a small animal containment and fed *ad libitum* with sterile water and certified mouse chow. All animal studies were approved by the Pasteur Institute Safety Committee (protocol 12.062) in accordance with French and European guidelines, and mice were cared for in accordance with the Pasteur Institute guidelines in compliance with the European Animal Welfare regulations.

### Intraperitoneal and intranasal inoculations.

Bacteria grown to the exponential phase (optical density at 600 nm [OD_600_] of 0.8 to 0.9) in BHI broth were centrifuged, resuspended in phosphate-buffered saline (PBS), and used immediately. To determine the intraperitoneal (i.p.) 50% lethal dose (LD_50_), five 3-fold dilutions from 2.0 × 10^6^ to 5.5 × 10^4^ CFU of strain BM4587 were injected i.p. to groups of six 7-week-old C57BL/6 female mice, and the mice were monitored for 7 days. All survivors showed a normal clinical score by 4 days postinoculation (p.i.) (see Fig. S1 in the supplemental material). Mice given the highest inoculum died in 1 day, and mice given the two lowest inocula survived until the end of the experiment. Survival rates of the mice given two intermediate inocula were 83.3% and 16.7%, respectively. The BM4587 i.p. LD_50_ value, calculated by probit analysis, was 4.9 × 10^5^ CFU/mouse. Unless otherwise specified, mice were inoculated i.p. with ca. 5 × 10^5^ *A. baumannii* in 100 µl of PBS.

For the intranasal (i.n.) route, groups of mice anesthetized by i.p. injection of a mixture of ketamine (Imalgène 1000; Merial) and xylazine (Rompun; Bayer) were inoculated with six inocula from 2.5 × 10^6^ to 1 × 10^8^ CFU in 50 µl of PBS by dropping the inoculum on the tip of the nose. The mice were clinically monitored and sacrificed by 4, 24, and 48 h. By 12 h, infected mice had ruffled fur but they were all alive, and all mice had recovered by 24 h. Bacteria were not recovered from the blood, spleen, or liver of mice at any time point, and bacterial load in the lungs declined by more than 2 log_10_ units between 4 h and 48 h. Five log_10_ units of CFU/lung were harvested by 48 h after an inoculum of 1 × 10^7^ CFU. Therefore, ca. 5 × 10^7^ of *A. baumannii* was used for i.n. inoculation.

For bacterial growth kinetics, mice in each group were euthanized by i.p. administration of a lethal dose of pentobarbital at the indicated times. Systemic blood was collected by cardiac puncture, and the lungs, liver, and spleen were aseptically removed, weighed, and homogenized in sterile PBS using a Glas-Col homogenizer. Aliquots of 10-fold serial dilutions of the homogenates were cultured on BHI agar to quantify the number of viable *A. baumannii* in the respective organs.

For *in vivo* competition, mice were inoculated with a 1:1 mixture of parental strain BM4587 and mutant strain BM4689[↗*adeABC*] (BM4689 overexpressing *adeABC*) at a total of ca. 5 × 10^5^ and 5 × 10^7^ bacteria for i.p. and i.n. inoculation, respectively. The proportion of resistant strains was determined by replica plating on plates containing gentamicin (12 µg/ml).

### Bronchoalveolar lavage fluid analysis.

The trachea of euthanized mice were cannulated, and the lungs were washed four times with 0.5 ml PBS, yielding ca. 2 ml of bronchoalveolar lavage fluid (BALF). Cytospin slides of ca. 1 × 10^4^ BALF cells were prepared using a cytospin centrifuge and stained with Hema 3 Stat (Fisher, Pittsburgh, PA). The total and differential numbers of neutrophils and macrophages were determined by examining 200 cells. Cell-free BALF obtained after centrifugation (1,500 rpm or 5,000 rpm for 10 min) was used for chemokine measurement. The level of myeloperoxidase (MPO) in BALF was measured by enzyme immunometric assay, and murine interleukin-6 (IL-6), tumor necrosis factor alpha (TNF-α), and keratinocyte chemoattractant protein (KC) in BALF were quantified by a sandwich enzyme-linked immunosorbent assay (ELISA) with Duoset ELISA development kits (R&D Systems, Minneapolis, MN) according to the manufacturer’s instructions.

### Histopathological examination.

Groups of five mice were inoculated i.n. or i.p. with *A. baumannii* BM4587 or BM4689[↗*adeABC*]. Mice were sacrificed by i.p. administration of a lethal dose of pentobarbital on 4, 24, and 48 h after inoculation. The lungs were perfused by intratracheal instillation of 0.5 ml of 4% neutral buffered formalin. The lungs were removed, and sections were taken, immediately fixed in 4% neutral buffered formalin, and routinely embedded in paraffin. The sections were 4 μm thick and stained with hematoxylin and eosin (H&E) or toluidine blue. All sections were examined by light microscopy. A semiquantitative scoring system was applied to evaluate the microscopic changes. The scores were as follows: 1 for minimal, 2 for mild, 3 for moderate, 4 for marked, and 5 for severe. Microphotographs (Fig. 5 and 6) are representative of the histological findings in each group.

### Statistical analysis.

Data are presented as means ± standard deviations (SD) or standard errors (SE) for each group as specified. Differences in quantitative measurement were assessed by Student’s *t* test or by two-way Mann-Whitney analysis of variance (ANOVA) followed by the Shapiro-Wilk test. A *P* value of <0.05 was considered significant.

## SUPPLEMENTAL MATERIAL

Figure S1  Mouse survival (A) and clinical score (B) during 4 days following i.p. infection. Groups of six C57BL/6 mice were challenged with five inocula of *A. baumannii* BM4587. Survival and clinical scores were monitored every 12 h for 4 days. Clinical signs of each mouse were scored according to the following criteria: 0 for no abnormal clinical signs; −1 for ruffled fur but lively; −2 for ruffled fur, activity level slowing, sick; −3 for ruffled fur, eyes squeezed shut, bunched, hardly moving, very sick; −4 for moribund; −5 for dead. Error bars present SD. Download Figure S1, DOCX file, 0.1 MB

## References

[1] Dijkshoorn L, Nemec A, Seifert H 2007 An increasing threat in hospitals: multidrug-resistant *Acinetobacter baumannii*. Nat Rev Microbiol 5:939–951. doi:10.1038/nrmicro1789. 18007677

[2] Coyne S, Courvalin P, Périchon B 2011 Efflux-mediated antibiotic resistance in *Acinetobacter* spp. Antimicrob Agents Chemother 55:947–953. doi:10.1128/AAC.01388-10. 21173183 PMC3067115

[3] Magnet S, Courvalin P, Lambert T 2001 Resistance-nodulation-cell division-type efflux pump involved in aminoglycoside resistance in *Acinetobacter baumannii* strain BM4454. Antimicrob Agents Chemother 45:3375–3380. doi:10.1128/AAC.45.12.3375-3380.2001. 11709311 PMC90840

[4] Coyne S, Rosenfeld N, Lambert T, Courvalin P, Périchon B 2010 Overexpression of resistance-nodulation-cell division pump AdeFGH confers multidrug resistance in *Acinetobacter baumannii*. Antimicrob Agents Chemother 54:4389–4393. doi:10.1128/AAC.00155-10. 20696879 PMC2944555

[5] Damier-Piolle L, Magnet S, Brémont S, Lambert T, Courvalin P 2008 AdeIJK, a resistance-nodulation-cell division pump effluxing multiple antibiotics in *Acinetobacter baumannii*. Antimicrob Agents Chemother 52:557–562. doi:10.1128/AAC.00732-07. 18086852 PMC2224764

[6] Marchand I, Damier-Piolle L, Courvalin P, Lambert T 2004 Expression of the RND-type efflux pump AdeABC in *Acinetobacter baumannii* is regulated by the AdeRS two-component system. Antimicrob Agents Chemother 48:3298–3304. doi:10.1128/AAC.48.9.3298-3304.2004. 15328088 PMC514774

[7] Rosenfeld N, Bouchier C, Courvalin P, Périchon B 2012 Expression of the resistance-nodulation-cell division pump AdeIJK in *Acinetobacter baumannii* is regulated by AdeN, a TetR-type regulator. Antimicrob Agents Chemother 56:2504–2510. doi:10.1128/AAC.06422-11. 22371895 PMC3346617

[8] Yoon EJ, Courvalin P, Grillot-Courvalin C 2013 RND-type efflux pumps in multidrug-resistant clinical isolates of *Acinetobacter baumannii*: major role for AdeABC overexpression and AdeRS mutations. Antimicrob Agents Chemother 57:2989–2995. doi:10.1128/AAC.02556-12. 23587960 PMC3697384

[9] Yoon EJ, Chabane YN, Goussard S, Snesrud E, Courvalin P, Dé E, Grillot-Courvalin C 2015 Contribution of resistance-nodulation-cell division efflux systems to antibiotic resistance and biofilm formation in *Acinetobacter baumannii*. mBio 6:e00697-16. doi:10.1128/mBio.00309-15. PMC4453527 25805730

[10] Rumbo C, Gato E, López M, Ruiz de Alegría C, Fernández-Cuenca F, Martínez-Martínez L, Vila J, Pachón J, Cisneros JM, Rodríguez-Baño J, Pascual A, Bou G, Tomás M, Spanish Group of Nosocomial Infections and Mechanisms of Action and Resistance to Antimicrobials (GEIH-GEMARA), Spanish Society of Clinical Microbiology and Infectious Diseases (SEIMC), Spanish Network for Research in Infectious Diseases (REIPI) 2013 Contribution of efflux pumps, porins, and beta-lactamases to multidrug resistance in clinical isolates of *Acinetobacter baumannii*. Antimicrob Agents Chemother 57:5247–5257. doi:10.1128/AAC.00730-13. 23939894 PMC3811325

[11] Andersson DI, Hughes D 2010 Antibiotic resistance and its cost: is it possible to reverse resistance? Nat Rev Microbiol 8:260–271. doi:10.1038/nrmicro2319. 20208551

[12] Piddock LJ 2006 Multidrug-resistance efflux pumps - not just for resistance. Nat Rev Microbiol 4:629–636. doi:10.1038/nrmicro1464. 16845433

[13] Nishino K, Latifi T, Groisman EA 2006 Virulence and drug resistance roles of multidrug efflux systems of *Salmonella enterica* serovar Typhimurium. Mol Microbiol 59:126–141. doi:10.1111/j.1365-2958.2005.04940.x. 16359323

[14] Hirakata Y, Srikumar R, Poole K, Gotoh N, Suematsu T, Kohno S, Kamihira S, Hancock RE, Speert DP 2002 Multidrug efflux systems play an important role in the invasiveness of *Pseudomonas aeruginosa*. J Exp Med 196:109–118. doi:10.1084/jem.20020005. 12093875 PMC2194012

[15] Padilla E, Llobet E, Doménech-Sánchez A, Martínez-Martínez L, Bengoechea JA, Albertí S 2010 *Klebsiella pneumoniae* AcrAB efflux pump contributes to antimicrobial resistance and virulence. Antimicrob Agents Chemother 54:177–183. doi:10.1128/AAC.00715-09. 19858254 PMC2798511

[16] Pérez A, Poza M, Fernández A, del Carmen Fernández M, Mallo S, Merino M, Rumbo-Feal S, Cabral MP, Bou G 2012 Involvement of the AcrAB-TolC efflux pump in the resistance, fitness, and virulence of *Enterobacter cloacae*. Antimicrob Agents Chemother 56:2084–2090. doi:10.1128/AAC.05509-11. 22290971 PMC3318359

[17] Warner DM, Folster JP, Shafer WM, Jerse AE 2007 Regulation of the MtrC-MtrD-MtrE efflux-pump system modulates the *in vivo* fitness of *Neisseria gonorrhoeae*. J Infect Dis 196:1804–1812. doi:10.1086/522964. 18190261

[18] Alonso A, Morales G, Escalante R, Campanario E, Sastre L, Martinez JL 2004 Overexpression of the multidrug efflux pump SmeDEF impairs *Stenotrophomonas maltophilia* physiology. J Antimicrob Chemother 53:432–434. doi:10.1093/jac/dkh074. 14739147

[19] Olivares J, Alvarez-Ortega C, Linares JF, Rojo F, Köhler T, Martínez JL 2012 Overproduction of the multidrug efflux pump MexEF-OprN does not impair *Pseudomonas aeruginosa* fitness in competition tests, but produces specific changes in bacterial regulatory networks. Environ Microbiol 14:1968–1981. doi:10.1111/j.1462-2920.2012.02727.x. 22417660

[20] McConnell MJ, Actis L, Pachón J 2013 *Acinetobacter baumannii*: human infections, factors contributing to pathogenesis and animal models. FEMS Microbiol Rev 37:130–155. doi:10.1111/j.1574-6976.2012.00344.x. 22568581

[21] de Breij A, Eveillard M, Dijkshoorn L, van den Broek PJ, Nibbering PH, Joly-Guillou ML 2012 Differences in *Acinetobacter baumannii* strains and host innate immune response determine morbidity and mortality in experimental pneumonia. PLoS One 7:e00697-16. doi:10.1371/journal.pone.0030673. PMC3275605 22347396

[22] van Faassen H, KuoLee R, Harris G, Zhao X, Conlan JW, Chen W 2007 Neutrophils play an important role in host resistance to respiratory infection with *Acinetobacter baumannii* in mice. Infect Immun 75:5597–5608. doi:10.1128/IAI.00762-07. 17908807 PMC2168347

[23] Wang N, Ozer EA, Mandel MJ, Hauser AR 2014 Genome-wide identification of *Acinetobacter baumannii* genes necessary for persistence in the lung. mBio 5:e00697-16. doi:10.1128/mBio.01163-14. PMC4049102 24895306

[24] Breslow JM, Meissler JJ Jr, Hartzell RR, Spence PB, Truant A, Gaughan J, Eisenstein TK 2011 Innate immune responses to systemic *Acinetobacter baumannii* infection in mice: neutrophils, but not interleukin-17, mediate host resistance. Infect Immun 79:3317–3327. doi:10.1128/IAI.00069-11. 21576323 PMC3147579

[25] López-Rojas R, McConnell MJ, Jiménez-Mejías ME, Domínguez-Herrera J, Fernández-Cuenca F, Pachón J 2013 Colistin resistance in a clinical *Acinetobacter baumannii* strain appearing after colistin treatment: effect on virulence and bacterial fitness. Antimicrob Agents Chemother 57:4587–4589. doi:10.1128/AAC.00543-13. 23836165 PMC3754302

[26] Roux D, Danilchanka O, Guillard T, Cattoir V, Aschard H, Fu Y, Angoulvant F, Messika J, Ricard JD, Mekalanos JJ, Lory S, Pier GB, Skurnik D 2015 Fitness cost of antibiotic susceptibility during bacterial infection. Sci Transl Med 7:297ra114. doi:10.1126/scitranslmed.aab1621. 26203082

[27] Lesho E, Yoon EJ, McGann P, Snesrud E, Kwak Y, Milillo M, Onmus-Leone F, Preston L, St Clair K, Nikolich M, Viscount H, Wortmann G, Zapor M, Grillot-Courvalin C, Courvalin P, Clifford R, Waterman PE 2013 Emergence of colistin-resistance in extremely drug-resistant *Acinetobacter baumannii* containing a novel *pmrCAB* operon during colistin therapy of wound infections. J Infect Dis 208:1142–1151. doi:10.1093/infdis/jit293. 23812239

[28] Touchon M, Cury J, Yoon EJ, Krizova L, Cerqueira GC, Murphy C, Feldgarden M, Wortman J, Clermont D, Lambert T, Grillot-Courvalin C, Nemec A, Courvalin P, Rocha EP 2014 The genomic diversification of the whole *Acinetobacter* genus: origins, mechanisms, and consequences. Genome Biol Evol 6:2866–2882. doi:10.1093/gbe/evu225. 25313016 PMC4224351

[29] Gebhardt MJ, Gallagher LA, Jacobson RK, Usacheva EA, Peterson LR, Zurawski DV, Shuman HA 2015 Joint transcriptional control of virulence and resistance to antibiotic and environmental stress in *Acinetobacter baumannii*. mBio 6:e00697-16. doi:10.1128/mBio.01660-15. PMC4659468 26556274

[30] Skurnik D, Roux D, Cattoir V, Danilchanka O, Lu X, Yoder-Himes DR, Han K, Guillard T, Jiang D, Gaultier C, Guerin F, Aschard H, Leclercq R, Mekalanos JJ, Lory S, Pier GB 2013 Enhanced *in vivo* fitness of carbapenem-resistant *oprD* mutants of *Pseudomonas aeruginosa* revealed through high-throughput sequencing. Proc Natl Acad Sci U S A 110:20747–20752. doi:10.1073/pnas.1221552110. 24248354 PMC3870709

[31] Marks M, Burns T, Abadi M, Seyoum B, Thornton J, Tuomanen E, Pirofski LA 2007 Influence of neutropenia on the course of serotype 8 pneumococcal pneumonia in mice. Infect Immun 75:1586–1597. doi:10.1128/IAI.01579-06. 17296760 PMC1865693

[32] Ramphal R, Balloy V, Jyot J, Verma A, Si-Tahar M, Chignard M 2008 Control of *Pseudomonas aeruginosa* in the lung requires the recognition of either lipopolysaccharide or flagellin. J Immunol 181:586–592. doi:10.4049/jimmunol.181.1.586. 18566425 PMC2504754

[33] Coyne S, Guigon G, Courvalin P, Périchon B 2010 Screening and quantification of the expression of antibiotic resistance genes in *Acinetobacter baumannii* with a microarray. Antimicrob Agents Chemother 54:333–340. doi:10.1128/AAC.01037-09. 19884373 PMC2798560

[34] Clinical and Laboratory Standards Institute 2015 Performance standards for antimicrobial disk susceptibility tests. M02-A12. Clinical and Laboratory Standards Institute, Wayne, PA.

[35] Foucault ML, Depardieu F, Courvalin P, Grillot-Courvalin C 2010 Inducible expression eliminates the fitness cost of vancomycin resistance in enterococci. Proc Natl Acad Sci U S A 107:16964–16969. doi:10.1073/pnas.1006855107. 20833818 PMC2947908

