# Supplementary material for: Contribution of the Ade Resistance-Nodulation-Cell Division-Type Efflux Pumps to Fitness and Pathogenesis of Acinetobacter baumannii
Source: mBio. 2016 May 31;7(3):e00697-16. doi: 10.1128/mBio.00697-16 (PMC4895114; doi:10.1128/mBio.00697-16)
Supplement: Figure S1 — Mouse survival (A) and clinical score (B) during 4 days following i.p. infection. Groups of six C57BL/6 mice were challenged with five inocula of A. baumannii BM4587. Survival and clinical scores were monitored every 12 h for 4 days. Clinical signs of each mouse were scored according to the following criteria: 0 for no abnormal clinical signs; −1 for ruffled fur but lively; −2 for ruffled fur, activity level slowing, sick; −3 for ruffled fur, eyes squeezed shut, bunched, hardly moving, very sick; −4 for moribund; −5 for dead. Error bars present SD. Download [file mbo003162830sf1.docx]

**FIG S1** Mice survival (A) and clinical score (B) during 4 days following i.p. infection. Groups of six C57BL/6 mice were challenged with five inocula of *A. baumannii* BM4587. Survival and clinical scores were monitored every 12 h for 4 days. Clinical signs of each mouse were scored according to the criteria: 0, no abnormal clinical signs; -1, ruffled fur but lively; -2, ruffled fur, activity level slowing, sick; -3, ruffled fur, eyes squeezed shut, bunched, hardly moving, very sick; -4, moribund; -5, dead. Error bars present SD.
